# The Emerging Roles and Therapeutic Implications of Epigenetic Modifications in Ovarian Cancer

**DOI:** 10.3389/fendo.2022.863541

**Published:** 2022-05-10

**Authors:** Yu Wang, Zhao Huang, Bowen Li, Lin Liu, Canhua Huang

**Affiliations:** ^1^ State Key Laboratory of Biotherapy and Cancer Center, West China Hospital, and West China School of Basic Medical Sciences & Forensic Medicine, Sichuan University, and Collaborative Innovation Center for Biotherapy, Chengdu, China; ^2^ Department of Anesthesiology, The Affiliated Hospital of Medical School, Ningbo University, Ningbo, China

**Keywords:** ovarian cancer, drug resistance, cancer epigenetics, DNA methylation, histone modifications, non-coding RNA, epigenetic therapy

## Abstract

Ovarian cancer (OC) is one of the most lethal gynecologic malignancies globally. In spite of positive responses to initial therapy, the overall survival rates of OC patients remain poor due to the development of drug resistance and consequent cancer recurrence. Indeed, intensive studies have been conducted to unravel the molecular mechanisms underlying OC therapeutic resistance. Besides, emerging evidence suggests a crucial role for epigenetic modifications, namely, DNA methylation, histone modifications, and non-coding RNA regulation, in the drug resistance of OC. These epigenetic modifications contribute to chemoresistance through various mechanisms, namely, upregulating the expression of multidrug resistance proteins (MRPs), remodeling of the tumor microenvironment, and deregulated immune response. Therefore, an in-depth understanding of the role of epigenetic mechanisms in clinical therapeutic resistance may improve the outcome of OC patients. In this review, we will discuss the epigenetic regulation of OC drug resistance and propose the potential clinical implications of epigenetic therapies to prevent or reverse OC drug resistance, which may inspire novel treatment options by targeting resistance mechanisms for drug-resistant OC patients.

## 1 Introduction

With around 239,000 new cases and 152,000 deaths each year, ovarian cancer (OC) is the seventh most prevalent cancer and the second leading cause of death from gynecologic cancer (1). Despite advances in surgical procedures, platinum-based chemotherapy, targeted medicines, and immunotherapy in recent decades, patients with OC still have a poor prognosis due to advanced and extensive disseminated tumors (2–4). The high death rate of OC is partly attributable to its extremely invasive growth pattern, which is hard to detect in early stages and frequently resistant to drugs (5, 6).

**Figure 1:**
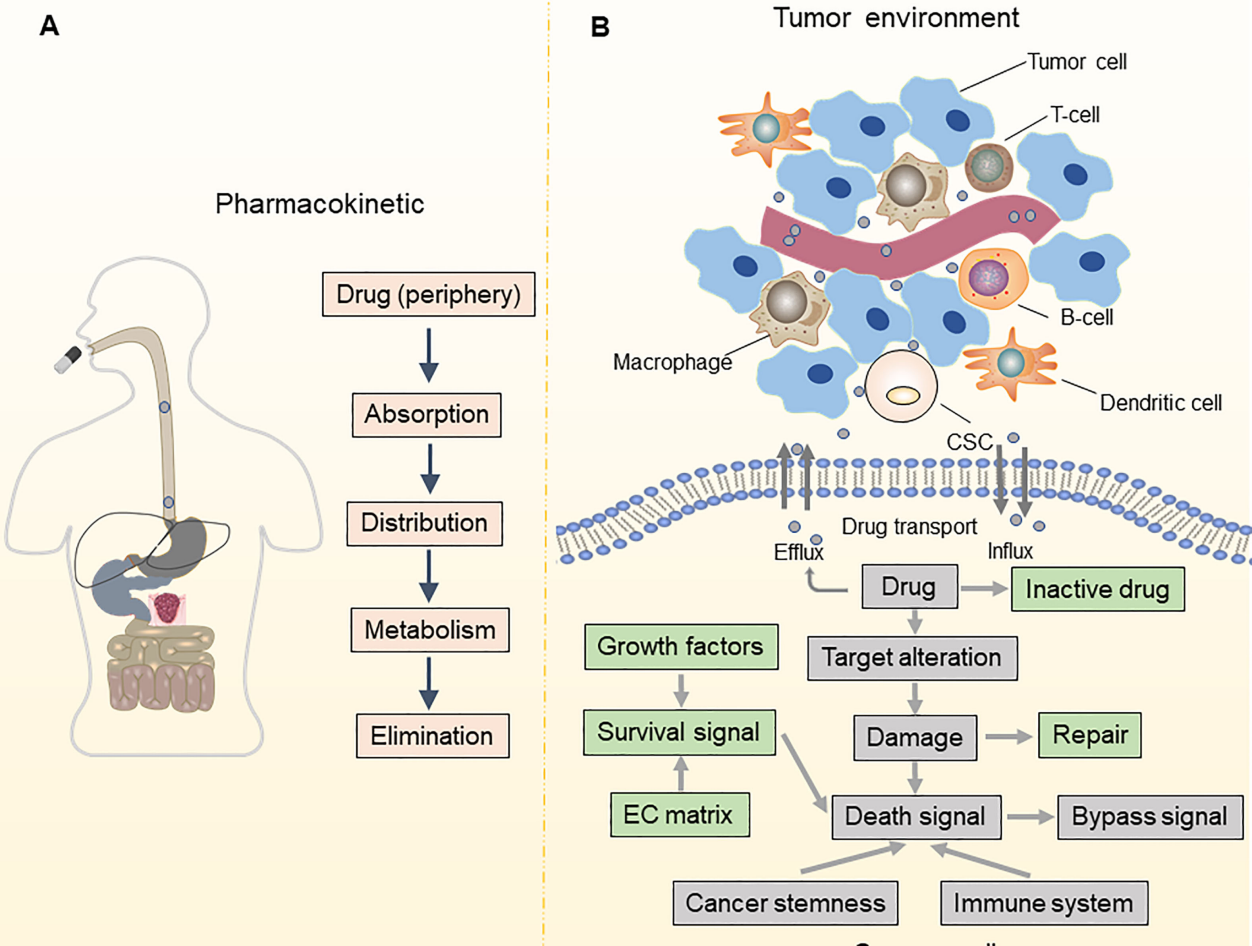
Pharmacokinetic and TME-associated factors contribute to drug resistance. **(A)** Mechanisms of drug resistance can be due to pharmacokinetics, including drug absorption, distribution, metabolism, and elimination; **(B)** Alterations in drug influx and efflux system impact the intracellular accumulation of anti-cancer drugs in tumor cells. The leading determinants of drug resistance are altered in drug targets, bypass signaling pathways, DNA damage and repair, and cell death signaling. Besides, maintenance of cancer stemness and a tumor-promoting immune microenvironment also contribute to drug resistance.

Drug resistance is a significant barrier to treating OC and leads to a poor prognosis. While 80% of individuals initially diagnosed with OC respond to conventional first-line therapy, such as platinum-based chemotherapy and surgical cytoreduction, roughly 75% of patients with advanced OC relapse within three years, which is usually fatal (2, 5). With notable therapeutic advantages, poly (ADP-ribose) polymerase (PARP) inhibitors have emerged as the first targeted medicines for patients with platinum-sensitive recurrent OC (7, 8). However, the effectiveness of PARP inhibitors is severely limited in OC due to the narrow spectrum of administration and various resistance mechanisms (9, 10). To improve the prognosis of patients with OC, it is vital to understand the underlying mechanisms of treatment resistance in OC and develop techniques to postpone or overcome drug resistance (**Figure 1**).

**Figure 2:**
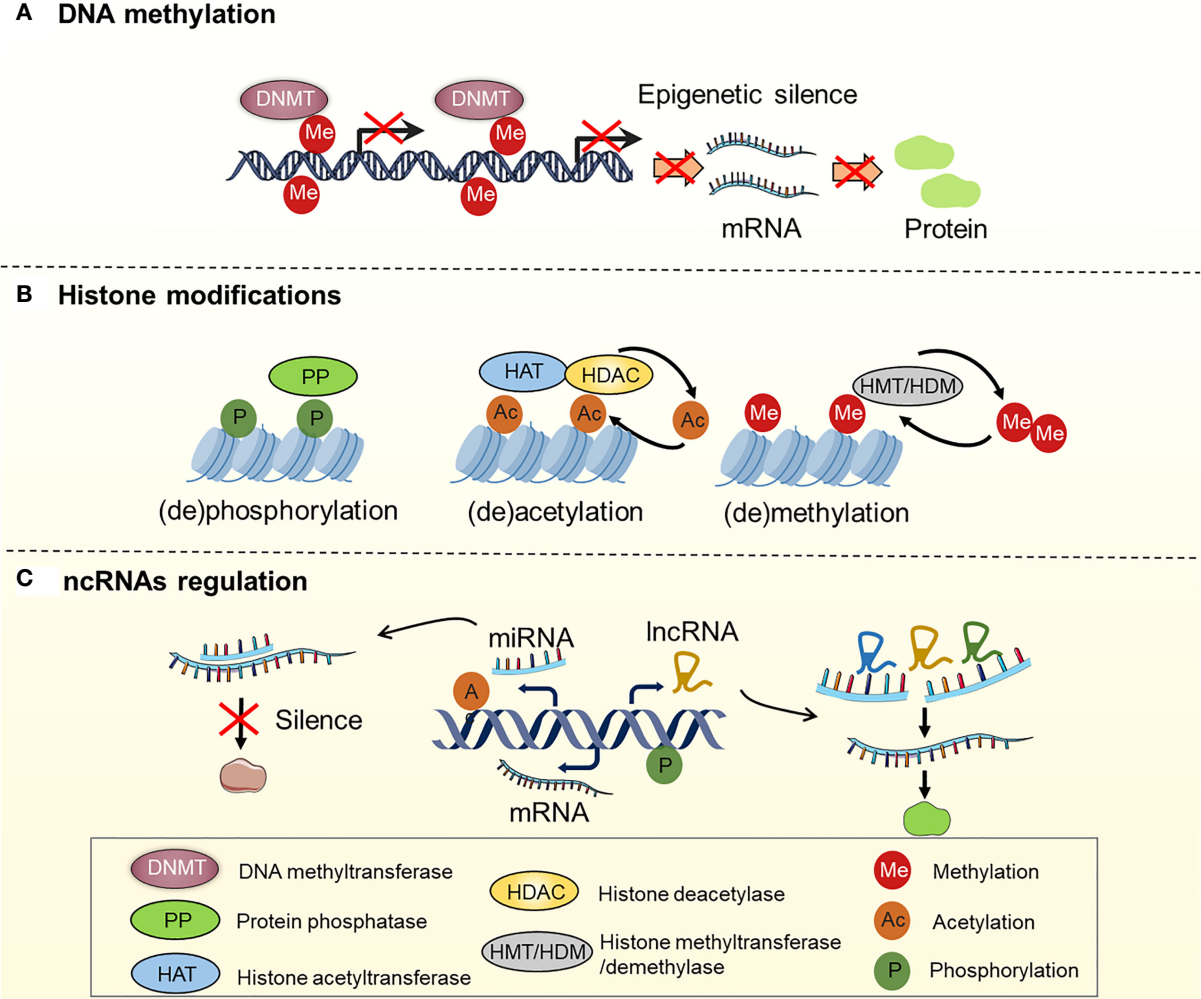
Regulation of DNA methylation, histone modifications and ncRNAs in OC. **(A)** Genes are silenced by hypermethylation, which is catalyzed by DNA methyltransferases (DNMTs); **(B)** Histone modifications, including histone (de)phosphorylation, (de)acetylation, (de)methylation, etc. Histone (de)phosphorylation is catalyzed by protein phosphatase (PP). Histone (de)acetylation is catalyzed by histone acetyltransferase (HAT) and histone deacetylase (HDAC). Histone (de)methylation is catalyzed by histone methyltransferase (HMT) and histone demethylase (HDM); **(C)** ncRNAs regulation: miRNAs and lncRNAs regulate gene expression by interacting with mRNA.

It is now generally accepted that drug resistance may arise from the diminished intracellular accumulation of drugs, alterations of drug targets, deregulation of immune response, and issues with the cell death executioner machinery, as well as the generation and maintenance of drug-resistant cells (11–15). Furthermore, a recent study suggested that epigenetic regulation (namely, DNA methylation, histone modifications, and non-coding RNA regulation) is one of the key mechanisms in OC that drives both intrinsic and acquired treatment resistance (16–19) (**Figure 2**). For instance, as one of the most effective broad-spectrum anti-cancer drugs, cisplatin kills tumor cells *via* DNA damage. However, epigenetic alterations are frequently observed in this process and are associated with cisplatin resistance (20, 21). For example, DNA methylation plays an indispensable role in OC drug resistance. Early studies suggested that hypermethylation of the BRCA1 gene in OC cells confers susceptibility to platinum-based chemotherapy (22, 23). At least 20 post-translational modifications occur in histone to govern the structures and activities of DNA. Accumulating evidence demonstrates that histone modifications, namely, acetylation, methylation, and phosphorylation, are linked to OC development and treatment resistance (24–26). Moreover, non-coding RNAs (ncRNAs), such as long non-coding RNAs (lncRNAs) and microRNAs (miRNAs), are currently recognized to be involved in various biological processes, including drug resistance (27, 28). As discussed above, we have summarized the epigenetic regulation in OC drug resistance (**Table 1**). Importantly, epigenetic alterations are reversible, and emerging epigenome-targeted therapy strategies can overcome OC drug resistance by reversing histone modifications and DNA methylation or by targeting ncRNAs. In this review, we will discuss the detailed mechanisms of epigenetic regulation that contribute to drug resistance in OC and highlight the advantages and challenges of epigenome-targeted therapy strategies for treating OC.

**Table 1:** Summary of epigenetic regulation in OC drug resistance.

Epigenetic regulation	Resistance against	Function	Target/pathway	References
MGMT	Paclitaxel Cisplatin	Chemoresistance	MGMT. DUB3, MCL1	(29)
DNMT1	Paclitaxel	Reverse paclitaxel resistance	DNMT1/CHFR/ Aurora A	(30)
DNMT3A	Cisplatin	Cisplatin resistance	miR-143	(31)
DNMT3B	Cisplatin	Autophagy, cisplatin resistance	RBP1	(32)
H3K14ac	Paclitaxel Cisplatin	Chemoresistance	RBP2/KDM5A/ Jarid1A	(33)
H3K27ac	Platinum	Platinum resistance	IL2/STAT5, TGF-β	(34)
miR-136	Paclitaxel	Inhibition of proliferation	Notch3	(35)
miR-98-5p	Cisplatin	Promotion of drug resistance	miR-98-5p/ Dicer1/miR-152	(36)
miR-142-5p	Cisplatin	Inhibition of drug resistance	XIAP, BIRC3, BCL2	(37)
miR-509-3p	Platinum	Enhance drug sensitivity	GOLPH3, WLS	(38)
miR-34a-5p	Cisplatin	Inhibition of proliferation	PD-L1	(39)
HOTAIR	Cisplatin	DNA damage response	NF-κB, miR-200c	(40, 41)
UCA1	Paclitaxel Cisplatin	Drug effluent system	miR-143/FOSL2	(42)
BC200	Carboplatin	Tumor suppressor	Proliferation	(43)
GAS5	Platinum	Induction of apoptosis	Cyclin D1, p21, APAF1	(44)
LSINCT5	Paclitaxel	Promotion of proliferation, migration, invasion	CXCL12/CXCR4	(45)

## 2 Epigenetic Modifications in OC Drug Resistance

Since the discovery of methylation of DNA repair genes by O6-methylguanine-DNA methyltransferase (MGMT), the role of epigenetic modifications in the context of inherent or acquired drug resistance has been extensively explored (46). Multiple mechanisms, namely, altered drug targets and bypass signaling pathways, enhanced drug efflux and metabolism, downstream adaptive responses, and maintenance of cancer stemness, are the primary causes of decreased anti-cancer drug effectiveness (47–50). Furthermore, emerging evidence suggests that the tumor microenvironment (TME) is crucial to multidrug resistance (MDR) in cancer cells (51–53). The molecular mechanisms of epigenetic regulation-mediated OC drug resistance, namely, enhanced drug efflux and metabolism, alteration of drug targets and bypass signaling pathways, downstream adaptive responses, maintenance of cancer stemness, and TME, are discussed here.

### 2.1 Epigenetic Modifications Involved in Drug Transport and Metabolism

**Figure 3:**
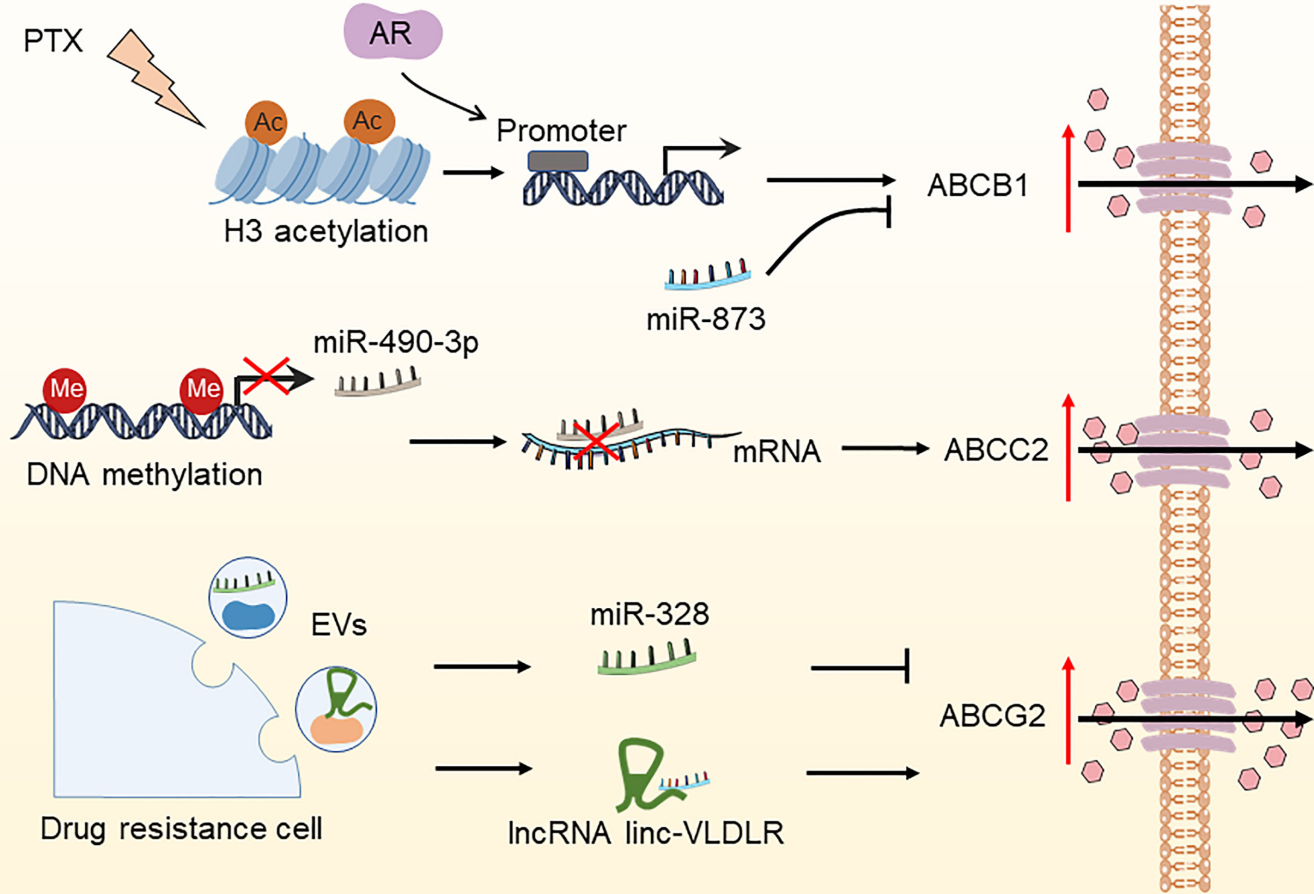
Epigenetic modifications regulate the expression of ABC transporters in OC. The DNA methylation of miR-490-3p, increased histone H3 acetylation due to paclitaxel treatment, miR-328, and lncRNA linc-VLDLR in EVs have been reported to increase drug efflux by regulating the expression of ABCC2, ABCB1, and ABCG2, thereby leading to OC drug resistance. PTX, paclitaxel; AR, androgen receptor; EVs, extracellular vesicles.

Effective cytotoxic drug treatment requires a sufficient intracellular drug concentration in tumor cells. Drug concentration is coordinated by transporters mediating the influx and efflux of drugs and enzymes mediating drug metabolism. The effectiveness of chemotherapeutic and targeted drug delivery into cancer cells is determined by drug inflow and efflux transporters. The most known such transporters are the ATP-binding cassette (ABC) transporter family members, namely, ABCB1 (MDR1), ABCC2 (MRP-2), and ABCG2 (BCRP/MXR1), which have been widely shown to be associated with drug resistance (54, 55). The epigenetic control of ABC transporter-induced OC resistance has recently progressed significantly **(** **Figure 3** **)**. ABCB1 is the first ABC transporter identified and plays a crucial role in determining drug sensitivity. The epigenetic regulation of ABCB1 is related to drug transportation in OC cells. For instance, paclitaxel therapy increased histone H3 acetylation and targeted the ABCB1 promoter in conjunction with the androgen receptor (AR), resulting in ABCB1 gene expression and the establishment of the paclitaxel resistance phenotype (56). Wu et al. found that overexpression of miR-873 improved the susceptibility of OC cells to paclitaxel and cisplatin by targeting ABCB1 (57). Besides, Tian et al. demonstrated that miR-490-3p enhances the sensitivity of OC cells to cisplatin by downregulating ABCC2 expression (58). Nevertheless, hnRNPA2B1 was shown to bind to the 5’UTR of ABCC2 mRNA and promote its translation, leading to cisplatin resistance in OC (59). Furthermore, ABCG2 is strongly expressed in cisplatin- or paclitaxel-resistant OC and OC stem cells, indicating the key role of ABCG2 in drug resistance and stemness acquisition in OC (60). Calcagno and colleagues discovered that elevated acetylation of histone H3 in the ABCG2 promoter is a cellular response to the treatment of doxorubicin, which underlies its doxorubicin resistance (61). Some lncRNAs and miRNAs found in extracellular vesicles (EVs) generated by drug-resistant cells control the expression of ABCG2, hence impacting tumor drug resistance (62–65). These investigations indicate that epigenetic regulation plays an essential role in OC drug resistance by modulating the ABC transporter family.

Drug metabolism regulates bioactivation, catabolism, conjugation, and excretion, determining drug clinical efficacy and toxicity (66). Drug metabolism enzymes can be affected by alterations in the expression level of affect drug metabolism. For instance, epigenetic alterations regulate the expression of the cytochrome P450 (CYP) enzyme family, which is the most well-known drug-metabolizing enzyme, thus affecting the metabolism of several anti-cancer medications. A recent study showed that histone modification enzyme G9a regulates the expression of CYP450 by affecting histone 3 lysine 4 and histone 3 lysine 27 methylation, suggesting G9a alters drug sensitivity (67). Luo and colleagues emphasized an unconventional epigenetic regulation mechanism of CYP gene expression; that is, miR-148a stimulates CYP2B6 expression by binding to the 3’UTR region to improve mRNA stability (68). Furthermore, miR-543 overexpression increases the production of CYP3A4, which is involved in the metabolism of ruxolitinib (69). Other drug-metabolizing enzymes are controlled by epigenetic alterations, which must be investigated further.

### 2.2 Epigenetic Modifications Regulate Drug Targets and Bypass Signaling Pathways

Over the past decade, targeted agents have been steadily introduced into clinical trials for treating recurrent OC, namely, the anti-VEGFR agent bevacizumab, PARP inhibitors (olaparib, niraparib, and rucaparib), the anti-MEK inhibitor trametinib, and anti-HER2 pertuzumab. However, chemotherapy resistance resulting from epigenetic alterations limits the effectiveness of these targeted treatments. Resistance to targeted therapy arises primarily through reduced expression of drug targets and activated bypass signaling pathways during treatment with targeted drugs (70–72). Here, we summarize the epigenetic modification-mediated drug resistance in OC, caused by the deregulated expression of drug targets, mutation of drug targets, and the persistent activation of bypass signaling pathways.

Angiogenesis is critical in the etiology of OC, which is associated with the generation of vascular endothelial growth factor (VEGF), linked to the progression of malignant ascites and OC (73, 74). In clinical trials, bevacizumab, an anti-VEGF monoclonal antibody, has been proven to improve outcomes in OC patients in clinical trials (75, 76). However, epigenetic modifications may diminish VEGF expression. Vesna and colleagues found twenty significant CpG sites in promoter regions, suggesting that the concentration of VEGF-A can be regulated by DNA methylation (77). Based on The Cancer Genome Atlas (TCGA) databases, Zhou and colleagues discovered that overexpression of insulin-like growth factor 2 mRNA-binding protein 3 (IGF2BP3) was related to cancer progression and poor survival of patients. Mechanistically, IGF2BP3 can bind with the mRNA of VEGF, where IGF2BP3 serves as a reader to recognize the m6A modification on VEGF mRNA. This effect controls both the production and stability of VEGF mRNA, which may affect drug sensitivity (78). Besides, VEGFA is the direct target of miR-652-5p, and miR-652-5p is down-regulated by hypermethylation at the upstream CpG site. Increasing VEGF synthesis is linked to tumor progression and metastasis (79). Moreover, it has been shown that cells with methylated BRCA1 have defective homologous recombination (HR) activity, thus being highly responsive to PARP inhibitors (olaparib, niraparib, and rucaparib), implying that BRCA1 inactivated by epigenetic mechanisms contributes to drug sensitivity (80–82).

In addition to alterations in drug targets, epigenetic modifications are implicated in the activation of bypass signaling pathways to regulate OC drug resistance. Although the Notch pathway is closely related to the growth of OC tumors, its clinical significance and molecular mechanisms remain unclear. Hu et al. found that alterations in the Notch pathway are prevalent and closely related to poor clinical outcomes in patients with OC (83, 84). For instance, epigenetic regulation of multiple Notch target genes (such as PPARG, CCND1, and RUNX1) can regulate the activation of the Notch pathway, which is associated with the prognosis of OC patients (85). Liu and colleagues found that Fas deficiency inhibits the release of miR-29b, thereby increasing intracellular miR-29b levels and subsequently downregulating DNA methyltransferase 1 (DNMT1) expression, which results in hypomethylation of the Notch1 promoter region and activation of Notch signaling (86). Hirsch et al. discovered that inhibition of histone deacetylase might alleviate abnormalities in the Notch and Eph axis in prion protein PrP deficient and prion-infected cells (87). Besides, studies have demonstrated that lncRNA HOTAIR induces OC drug resistance to cisplatin through activating the Wnt/β-catenin pathway (88). Further studies of epigenetic modifications that regulate drug targets and bypass signaling pathways may increase our understanding of the development of potential strategies to reverse drug resistance.

### 2.3 Epigenetic Modifications Modulate DNA Damage and Repair

Most chemotherapeutic drugs induce cell death through apoptosis due to DNA damage (89, 90). After treatment with cytotoxic agents, eukaryotic cells usually undergo damage repair to avoid apoptosis, leading to drug resistance (91). Therefore, components of the DNA repair system, such as O6-methylguanine-DNA methyltransferase (MGMT), can promote chemotherapeutic resistance. MGMT mediates the direct removal of O6-methylguanine (O6-MEG), a mark of DNA damage induced by temozolomide (TMZ), thereby facilitating DNA repair in melanoma cells. Other TMZ-induced lesions are mostly repaired by base excision repair (BER) or direct removal mechanisms catalyzed by DNA demethylase ALKBH2/3 (91, 92). Poly-ADP-ribose polymerase inhibitors (PARPis) are the most effective therapies approved for treating OC, and poly-ADP-ribose polymerase (PAPR) inhibitors (olaparib, niraparib, and rucaparib) are already being investigated in OC clinical trials. Indeed, all PARPis exhibit radio- or chemo-potentiation effects *in vitro* and *in vivo*, which is consistent with their ability to inhibit DNA damage repair (93, 94). According to a recent study by Nephew and colleagues, platinum-induced DNA damage contributes to the activation of the NF-κB pathway by the lncRNA HOTAIR and cellular senescence. Furthermore, DNA damage response activated NF-κB, which in turn triggered HOTAIR and created a positive-feedback loop, resulting in sustained NF-κB activation and persistent DNA damage signaling (40). Mutations in p53 are observed in 42% of human tumors (95). The regulation of RNA m6A on TP53 has been proven to overcome drug resistance by controlling downstream pathways and DNA damage repair, suggesting that it is a viable therapeutic approach (96). Meng et al. discovered that AZD1775, not only carboplatin, can increase sensitivity to gemcitabine and olaparib in TP53-mutated gynecologic cancer cells (97). WEE1 is a tyrosine kinase that blocks the CDK1/cyclin B complex by inducing CDK1 phosphorylation at tyrosine 15 (Y15), inducing cell cycle arrest and allowing DNA repair (98, 99). AZD1775 is a first-in-class, powerful, and selective WEE1 inhibitor that has shown a considerable anti-tumor effect in OC patients with TP53 mutations when combined with carboplatin (100). In conclusion, uncovering the mechanisms that influence epigenetic modification function in DNA damage repair may develop new strategies for the sensitization of OC cells to DNA damage inducers.

### 2.4 Epigenetic Modifications Activate Downstream Adaptive Responses

The interaction of anti-cancer drugs with corresponding cellular targets can induce cell death. However, a variety of adaptive cellular responses governed by epigenetic modifications are engaged to support the survival of tumor cells, which is the major target of cancer therapy. These include inhibition of apoptosis, initiation of autophagy, ferroptosis, and activation of pro-survival signals (101–103).

#### 2.4.1 Apoptosis

After medication therapy, cancer cells with enhanced DNA repair capacities can survive drug-induced DNA damage. The anti-apoptotic proteins (Bcl-2), inhibitor of apoptosis proteins (IAPs), and FLICE inhibitor proteins (FLIP) are upregulated in tumor cells, which contributes to their medication resistance (90). As an anti-apoptotic member of the Bcl-2 family, MCL1 plays an important role in the advanced chemotherapy resistance of OC. Wu et al. found that the deubiquitinating enzyme 3 (DUB3) in the cytoplasm of OC cells interacts with and deubiquitinates MCL1, thereby protecting MCL1 from degradation. Furthermore, they discovered that histone deacetylase inhibitors (HDACis) could increase the expression level of MGMT/DUB3, and HDACis combined with PaTrin-2 treatment has a significant effect on OC (29). Zhu et al. found that ALKBH5 is a potential target for OC therapy, which activates the EGFR-PIK3CA-AKT-mTOR signaling pathway, enhancing the stability of BCL-2 mRNA and promoting the interaction between Bcl-2 and Beclin1 (104). Abedini et al. discovered that p53 could promote the ubiquitination and subsequent proteasomal degradation of FLIP in response to cisplatin treatment, leading to the apoptosis of OC cells, potentially improving the therapeutic effects of cisplatin on OC.

#### 2.4.2 Autophagy

Autophagy partly enables tumor cells to cope with external stress, leading to the survival of cancer cells treated with anti-cancer drugs (105–107). Bi et al. have demonstrated that blocking autophagy can overcome resistance to HDAC inhibitors in gynecologic cancers (108). Autophagy can be activated by the upregulation of autophagy-related gene 14 (ATG14), and the abnormal expression of autophagy-related proteins contributes to drug resistance in OC. MiR-29c-3p inhibits autophagy and cisplatin resistance in part by downregulating the FOXP1/ATG14 pathway, suggesting that miR-29c-3p is a novel target for overcoming cisplatin resistance in OC (109). Bi et al. found that inhibition of methyltransferase-like 3 (METTL3) inhibits miR-126-5p’s upregulation of PTEN by regulating m6A modification, thereby preventing the activation of the PI3K/Akt/mTOR pathway and inhibiting the occurrence of OC. This finding highlights the role of m6A modification as a potential target for future OC treatment (110). O-Glcnacylation is a post-translational modification in which the O-GlCNAc transferase (OGT) transfers glucosamine (GlcNAc) to serine or threonine residues. Zhou et al. found that the levels of O-GlCNAC and OGT in OC chemically sensitive tissues were significantly higher than those in chemoresistant OC tissues, which reduced apoptotic cell death, resulting in increased resistance of OC cells to cisplatin (107). Together, the epigenetic modifications play an orchestrated role in regulating autophagy, and the effects of the epigenetic modifications on autophagy are cancer context-dependent. Additional studies are needed to elucidate other determinants of autophagy regulation related to epigenetic modifications.

#### 2.4.3 Ferroptosis

Using anti-cancer drugs to trigger apoptotic cell death is one of the principal ways to kill cancer cells. However, due to the acquired or intrinsic resistance of cancer cells to apoptosis, the effect of apoptosis inducers is limited (111, 112). Recently, a growing number of compounds and anti-cancer drugs kill tumor cells by ferroptosis (113–116). For instance, PARP inhibitors like olaparib kill BRCA mutant OC cells through ferroptosis (117). Ferroptosis is a newly discovered form of oxidative cell death caused by iron-dependent peroxidation of lipids. Thus, the redox balance controlled by various redox-active enzymes, which detoxify free radicals and lipid oxidation products, is critical for cells to avoid ferroptosis (118). Several regulatory molecules, such as GPX4, Nrf2, and members of the solute carrier (SLC) family of molecules, play essential roles in the aberrant iron metabolism and maladjustment of the antioxidant system. Studies have shown that epigenetic modification of these potent genes allows cancer cells to escape drug-induced ferroptosis, leading to drug resistance. Among them, ROS scavenger GPX4 is a key regulatory molecule of ferroptosis, which can convert lipid hydrogen peroxide into non-toxic lipid alcohols (119, 120). Due to upstream DNA hypomethylation and high levels of H3K27ac and H3K4me3, the expression level of GPX4 in OC tissues is higher than that in normal tissues, and it is negatively correlated with the prognosis of patients, indicating that aberrant GPX4 expression in cancer may result from epigenetic regulation (121). Additionally, they found that the GPX4 inhibitor RSL3 improves the anti-cancer effects of cisplatin by enhancing ferroptosis *in vitro* and *in vivo* (121, 122). Studies have shown that the activity of GPX4 depends on glutathione produced by system Xc (also known as SLC7A11 or xCT) (123). Indeed, p53 or the histone deubiquitinating enzyme BAP1 inhibiting SLC7A11 expression promotes ferroptosis (124, 125).

In addition to the above, the epigenetic alterations of transcription factor nuclear factor erythroid 2-related factor 2 (NRF2), a vital regulator of cellular antioxidant response and ferroptosis by upregulating SLC7A11, can affect the treatment resistance of OC (126, 127). For instance, hypermethylation of the gene promoter in the KEAP1/NRF2 axis has been described in various tumor tissues and is closely related to tumor recurrence and drug resistance (128–130). Van Jaarsveld et al. demonstrated that miR-141-mediated regulation of the KEAP1/NRF2 axis plays a crucial role in the OC response to cisplatin (131). Taken together, these findings provide evidence that epigenetic modifications play a significant role in ferroptosis and drug resistance of OC cells.

#### 2.4.4 Pro-Survival Signaling

Cell survival and death are determined by the epidermal growth factor receptor (EGFR) and AKT (protein kinase B) signaling pathways. Cell survival and death (132, 133) are regulated by HR and non-homologous terminal junctions (NHEJ). The activation of EGFR and AKT can be induced by epigenetic alterations, which may lead to chemotherapy resistance in OC treatment. Overexpression or gene amplification of EGFR and HER2 is frequent in multiple cancers, including OC (134). Cao et al. found that EGFR, phosphorylated EGFR (P-EGFR), and phosphorylated AKT (P-Akt) were up-regulated in miR-125A-5p- and TAZ-transfected OC cells through m (6) A modification (135). Lin et al. discovered that the RNA methyltransferase METTL3, which is involved in mRNA biosynthesis, degradation, and translation regulation, enhances the translation of EGFR and the TAZ in human cancer cells (136). Furthermore, Luo et al. have identified that the RNA polymerase II transcriptional mediator subunit 12 (MED12) down-regulates EGFR expression by binding to the EGFR promoter and mediates chemotherapy resistance in OC (137). Therefore, targeting epigenetic modifications might be an effective strategy to sensitize tumor cells to DNA-damaging agents.

### 2.5 Epigenetic Modifications Involved in the Maintenance of Cancer Stem Cells

Cancer stem cells (CSCs) are a sub-population of tumor cells that are responsible for driving tumor growth, metastasis, and therapy resistance (52, 138, 139). Emerging results indicate that CSCs contribute to chemoresistance and poor clinical outcomes in various malignancies, including OC (140). Accumulating evidence has shown that CSCs display an innate predisposition to be chemotherapy-resistant and result in tumor relapse (141–143). Furthermore, ovarian cancer stem cells (OCSCs) contribute to resistance to chemotherapy (144). Therefore, solving this thorny problem might lead to the development of new strategies to tackle drug-resistant OC. Because epigenetic regulation plays an integral role in the control of normal stem cell differentiation, strategies to target cancer stem cells can be developed through epigenetics. Epigenetic modifications enable cancer cells to self-renew and generate CSCs, leading to drug resistance (140).

Wang and colleagues hypothesized that hypomethylation drugs might target resistant OCSCs to cure tumors along with chemotherapy drugs. In an orthotopic mouse model, they identified and analyzed ALDH (+) OCSC from OC cell lines and clinical samples, finding that ALDH (+) cells were more chemoresistant than ALDH (−) cells. Treatment with SGI-110, a second-generation DNA methyltransferase inhibitor (DNMTi), re-sensitizes OCSCs to platinum (140). Xu et al. demonstrated that STON2 could negatively regulate the stemness in OC cells by DNMT1-MUC1. STON2 plays a role in OCSC biology and could be used as a therapeutic target for OC treatment (145). Studies have shown that MYPT1 encoding myosin phosphatase target subunit 1 is down-regulated in OC, leading to resistance to platinum-based therapy (146). Similarly, miR-30b could target MYPT1 to lead to enhanced CSC-like properties in OC cells and activate the Hippo pathway. Moreover, inhibition of YAP sensitizes cells to platinum-based therapy (50). Taken together, these studies suggest that epigenetic modifications of these signaling pathways play an essential role in the development of drug resistance in OC.

### 2.6 Epigenetic Modifications and the Tumor Microenvironment

**Figure 4:**
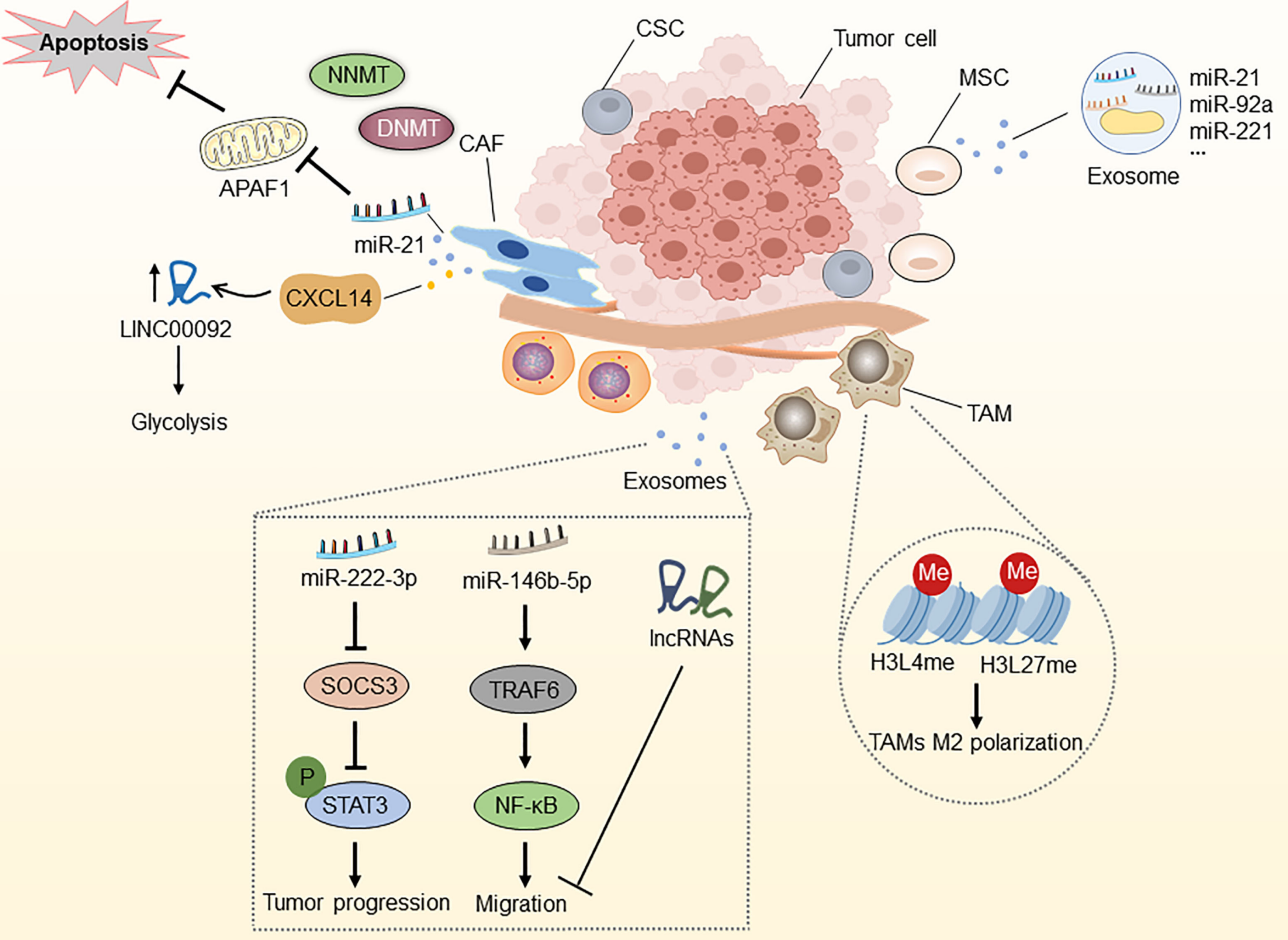
Epigenetic modifications in the tumor microenvironment. NNMT can diminish histone methylation in CAFs, and CAFs secrete miR-21 and CXCL14, which enhance OC development, metastasis, and therapy resistance. MiR-21, miR-92a, and miR-221, which are abundant in MSC-derived exosomes, are associated with OC development. Epigenetic alterations in histone H3 lysine4 and histone H3 lysine27 methylation in TAMs can induce macrophage M2 polarization. Epithelial ovarian cancer (EOC)-released exosomal miRNAs and lncRNAs *via* the SOCS3/STAT3 pathway and miR-146b-5P/TRAF6/NF-κB/MMP2 pathway regulate the progression, migration and drug resistance of OC. CAF, cancer-associated fibroblast; MSC, mesenchymal stem cell; TAM, tumor-associated macrophage; CSC, cancer stem cell; NNMT, nicotinamide N-methyltransferase; DNMT, DNA methyltransferase; APAF1, apoptosis protease activator-1; APAF6, TNF receptor associated factor 6.

A tumor microenvironment (TME) refers to the ecological niche in which tumor cells interact with the host stroma, including various immune cells, endothelial cells, fibroblasts, tumor cells, and metabolites. It is now generally accepted that TME significantly influences the efficacy of anti-cancer drugs (51). TME reprogramming caused by epigenetic dysregulations has recently been recognized as an important factor in the progression and drug resistance of OC (2, 147). Here, we systematically generalize the epigenetic modifications of non-malignant OC-related microenvironment cells (namely, cancer-associated fibroblasts, mesenchymal stem cells, and tumor-associated macrophages), which is conducive to evaluating the therapeutic potential of epigenetic regulation of TME-associated cells **(** **Figure 4** **)**.

#### 2.6.1 Cancer-Associated Fibroblasts (CAFs)

Fibroblasts located in the TME, also termed CAFs, are the main components of host stromal cells and the main source of collagenous cells in solid tumors (148). They play an essential role in supporting tumors by reconstructing the extracellular matrix. Multiple studies have identified the important role of CAFs in tumor-stroma communication through the excretion of various growth factors and chemokines, contributing to tumor growth, immunosuppression, angiogenesis, cell stemness, and drug resistance (149, 150). Therefore, epigenetic modifications associated with CAFs may affect the resistance of OC.

Studies have reported that the DNA methylation state of genes in mesenchymal fibroblasts from various cancer tissues conforms to the methylation features identified in nearby malignant cells (151, 152). Furthermore, the expression of stromal nicotinamide N-methyltransferase (NNMT) is essential for the functional aspects of the CAF phenotype and supports the growth and metastasis of OC. Stromal NNMT expression in CAFs depletes S-adenosyl methionine and histone methylation, which is related to the global alternations of gene expression in the tumor stroma (153). Additionally, CAFs secrete miR-21, which targets apoptosis protease activator-1 (APAF1), leading to paclitaxel resistance in metastatic or recurrent OC (154). New evidence suggests that OC cells reprogram fibroblasts into CAFs through the action of miRNAs in the TME, including miR-31, miR-214, and miR-155. Targeting these miRNAs in stromal cells might be of therapeutic value, suggesting these miRNAs as novel therapeutic targets for halting OC progression (155). Curtis and colleagues discovered that CAF-secreted CXCL14 promoted OC development and invasion by interacting with the major restriction enzyme, 6-Phosphofructo-2-Kinase/Fructo-2,6-Biphosphatase 2 (PFKFB2) and boosting LINC0009, a long non-coding RNA, to accelerate glycolysis (156, 157). These observations suggest that molecular insights into the abnormally expressed lncRNAs in CAFs are essential for the further diagnostic and therapeutic strategies of OC.

#### 2.6.2 Mesenchymal Stem Cells (MSCs)

MSCs contain various pluripotent cell subpopulations, and MSCs have been reported in most organs and tissues, including the ovaries (158). Because of their potential to develop into other active cancer-promoting stromal components (such as CAFs and cancer-associated adipocytes) and sustain cancer stem cell (CSC) populations, MSCs are strongly linked to cancer progression. In particular, OC-associated MSCs exhibit pluripotency and promote stem cell growth, increase resistance to platinum-based chemotherapy, and provide tumor matrix support and neovascularization (159–161). Although the great bulk of research has concentrated on understanding the impact of cellular signaling pathways, the epigenetic interactions between MSCs and OC remain largely unexplored.

Considering the function of epigenetic modifications in CSC reprogramming, MSCs can be efficiently transformed into CSCs by custom chromatin remodeling. Besides, MSCs can also differentiate into distinct stromal cells by epigenetic regulation (162–164). Through exosomal RNA sequencing, Reza et al. demonstrated numerous miRNAs that exhibit anti-cancer activity by targeting different molecules associated with OC survival (165). New evidence suggests that miR-21, miR-92a, and miR-221, abundant in MSC-derived extracellular vesicles, are associated with OC development (166). Taken together, the above findings revealed a direct or indirect epigenetic relationship between MSCs and OC cells, which should be investigated further and might lead to the identification of new therapeutic targets for OC.

#### 2.6.3 Tumor-Associated Macrophages (TAMs)

TAMs are the most abundant myeloid cell type in the TME and are involved in cancer-related inflammation, matrix remodeling, angiogenesis, metastasis, cancer cell stemness, tumor immune escape, and drug resistance (167–170). The unique TME determines macrophage diversity and the ability to switch between M1 (pro-inflammatory with anti-tumor activity) and M2 (anti-inflammatory with pro-tumor activity) phenotypes (171, 172). Epigenetic regulation plays a significant role in TAM differentiation and activation, which is significantly associated with tumor drug resistance. For instance, studies have found that epithelial ovarian cancer (EOC)-released exosomal miR-222-3p activated M2 polarization and tumor-promoting capacities in ovarian TAMs by the SOCS3/STAT3 pathway. Similarly, exosomal miR-940 released from hypoxic epithelial ovarian tumors induces a shift in the macrophage M2 phenotype (173, 174). Furthermore, TAM-secreted exosomes prevented endothelial migration by targeting the miR-146b-5P/TRAF6/NF-κB/MMP2 pathway, and the lncRNAs in OC-secreted exosomes effectively inhibit migration (175). Ishii et al. described M2 macrophage polarization through STAT6-mediated reciprocal epigenetic alterations in histone H3 lysine4 and histone H3 lysine27 methylation, which resulted in transcriptional activation of particular M2 manufacturing genes (176). Together, these findings highlight the importance of TAM-regulated epigenetic alterations in OC, as they may reveal novel diagnostic and therapeutic approaches.

## 3 Therapeutic Targeting of the OC Epigenome

**Figure 5:**
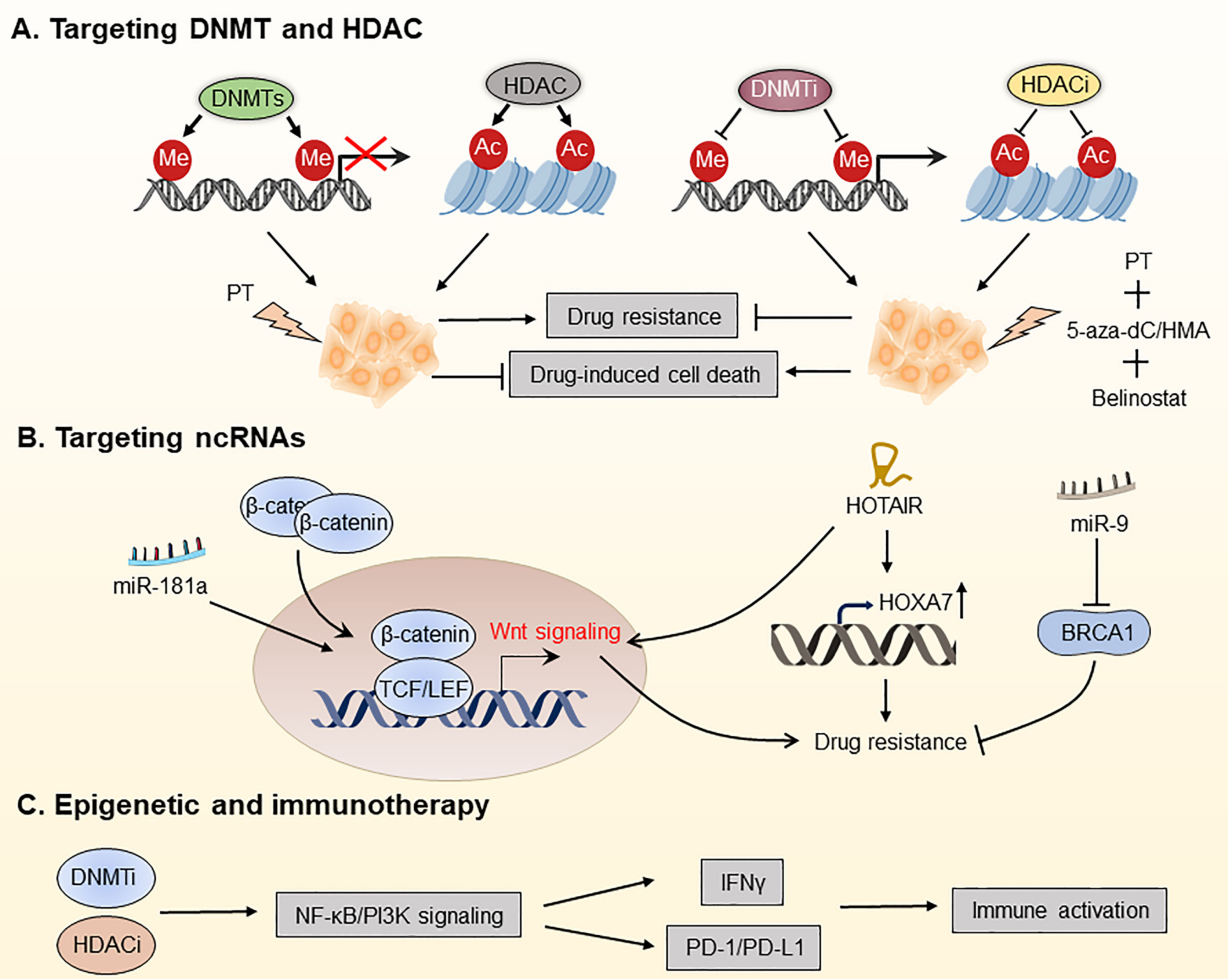
Targeting the OC epigenome and overcoming drug resistance in OC. **(A)** Platinum therapy combined with DNA methyltransferase inhibitors (DNMTis) and histone deacetylation inhibitors (HDACis) has been used for chemotherapeutic sensitization. **(B)** Overcoming OC drug resistance by targeting certain ncRNAs. **(C)** DNMTi and HDACi increased the responsiveness of OC to immune checkpoint therapy by reducing the immunosuppressive milieu *via* the type I IFN signaling pathway. PT, platinum; 5-aza-dC, 5-aza-2-deoxy-cytidine; HMA, hypomethylating agent; PD-1, programmed death protein 1; PD-L1, programmed death ligand 1.

OC treatment prioritizes surgery and cytoreduction, followed by cytotoxic platinum and taxane chemotherapy. Although most OC patients respond effectively in the early phases of therapy, new therapeutic options are urgently needed to enhance outcomes for the most advanced patients (1). In the last decade, immunotherapy and targeted therapy have shown remarkable benefits for treating OC (177–179). Furthermore, due to the reversibility of epigenetic alterations, the prevention of treatment resistance through epigenetic drugs has become an attractive therapeutic concept, and the combination of epigenetic therapies with chemotherapy, immunotherapy, and molecularly targeted treatments holds a lot of promise for OC treatment. Based on this, epigenetic regulation, namely, DNA methylation, histone modifications, and ncRNA regulation, has emerged as a potential therapeutic target for OC **(** **Figure 5** **)**.

### 3.1 Targeting DNMT

As one of the most well-known epigenetic modifications, DNA methylation plays an essential role in DNA repair, apoptosis, angiogenesis, gene expression regulation, and drug resistance. To date, aberrant DNA hypermethylation has been found in drug-resistant cancer cells, and drug-induced DNA hypermethylation has been proposed as a potential mechanism and biomarker of drug resistance. DNA methylation is mediated by three DNA methyltransferases (DNMTs), primarily by DNMT1, which mediates maintenance (one strand) methylation, and by DNMT3A and DNMT3B, which catalyze *de novo* methylation (180). The activity of DNMTs can be blocked by DNMT inhibitors (DNMTis), which are analogs of deoxycytosine and effectively block methyl transfer (181). 5-aza-2-deoxy-cytidine (decitabine) and 5-azacytidine were first successfully studied in hematological malignancies and myelodysplastic syndrome (MDS) and were approved by the FDA in 2006 for treating MDS, where they exhibit anti-tumor activity through the inhibition of DNA methylation (182).

To date, DNMTis has been successfully used for treating chemotherapy-resistant OC, restoring platinum sensitivity in refractory to standard chemotherapy of patients. DNA hypomethylation caused by DNMTi decitabine renders OC patients more susceptible to platinum treatment and is associated with a better prognosis. Matei et al. found that platinum resistance is mostly induced by epigenetic abnormalities, including abnormal DNA methylation. The regimen consists of a modest dosage of decitabine 5 days before the administration of carboplatin, which decreases toxicity and boosts the demethylation effect of decitabine, leading to the recovery of carboplatin sensitivity in patients with advanced OC (183). Decitabine has been shown in studies to be more effective than 5-azocytidine for treating platinum-resistant OC. It is capable of regulating the methylation status of the tumor antigen NY-ESO-1 to improve immunotherapy efficacy (184). Furthermore, decitabine improves responses of OC patients to platinum therapy by influencing signaling pathways that promote tumor progression, such as the TGF-β signaling pathway (185). Taken together, DNMTis plays a promising role in reversing or preventing chemotherapeutic and molecular-targeted drug resistance in OC patients.

### 3.2 Targeting HDAC

Histone deacetylase (HDAC) enzymes, which remove acetyl groups from histone and non-histone proteins, downregulate the transcription of genes (186). In cancer cells, HDAC inhibitors (HDACis) can restore transcriptional inhibition of tumor suppressor genes and generate an anti-cancer environment. Meng et al. showed that a combination of proteasome and HDAC inhibitors can inhibit gynecologic cancer growth (187). Inhibitors of enzyme-catalyzed histone modifications, among which HDACis are the most rapidly developed, have been investigated in solid tumors (188).

Fukumoto et al. found that ARID1A mutations confer sensitivity to pan-HDAC inhibitors such as SAHA in OC, which is associated with more significant effects of growth inhibition owing to suppression of HDAC2 activity (189). By suppressing HDAC6 with the small molecule ACY1215, Bitler et al. revealed that mice with ARID1A mutant tumors had dramatically increased survival. Mechanistically, HDAC6 deacetylates Lys120 on p53, thereby inactivating the pro-apoptotic function of p53 (190). However, not all HDAC subtypes are abnormally expressed in all malignancies, so pan-inhibition of HDAC is not an effective way to treat cancer (191). Besides, because of its poor activity as a single agent, HDACis have been investigated along with radiotherapy, chemotherapy, and other epigenetic drugs. For instance, belinostat re-sensitized drug-resistant OC cells to platinum, and the combination of decitabine and belinostat was more effective in re-sensitizing platinum than belinostat alone (192, 193). Furthermore, there is mounting evidence showing that HDACs-mediated deacetylation of non-histones is involved in many key cellular processes associated with drug resistance, such as apoptosis, suggesting that enhanced acetylation of non-histones using HDACis is promising for overcoming the drug resistance of OC. Taken together, targeting HDACs is an appealing strategy for OC treatment.

### 3.3 Targeting ncRNAs

Multiple ncRNAs, including miRNAs and lncRNAs, are increasingly implicated in the treatment resistance in OC (194, 195). Therefore, targeting tumor-specific miRNAs and lncRNAs is expected to overcome drug resistance of OC. Recently, miRNAs have received increasing attention as biomarkers and therapeutic targets for OC. For instance, Belur Nagaraj and colleagues found that miR-181a is an activator of Wnt/β-catenin signaling, driving stemness and chemotherapy resistance in high-grade serous ovarian cancer (HGSOC), thus being a potential target for treating recurrent OC (194). Using next-generation sequencing (NGS) techniques, Au Yeung et al. identified miR-21 in exosomes and tissue lysates isolated from cancer-associated adipocytes and fibroblasts, which is significantly higher than OC cells. They discovered that miR21 is transferred from CAAs or CAFs to OC cells, suppresses apoptosis and confers chemotherapeutic resistance in OC cells by binding with APAF1 (154). Additionally, Vescarelli et al. have demonstrated that miR-200c significantly enhanced the anti-cancer effect of the PARP inhibitor olaparib in drug-resistant OC cells (196). This finding indicates that combining olaparib with miRNA-based therapy is a potential therapeutic option for drug-resistant OC. Sun et al. found that miR-9 increases the sensitivity of OC cells to DNA damage by down-regulating BRCA1, thus improving the efficacy of chemotherapy (197).

In addition to miRNAs, LncRNAs are considered promising therapeutic targets for OC. For instance, the non-coding RNA HOTAIR has been shown to be a potential target for overcoming carboplatin resistance in OC (195). Furthermore, it has been discovered that HOTAIR is overexpressed in cisplatin-resistant OC cells, and knocking down HOTAIR enhances apoptosis in cisplatin-resistant OC cells by down-regulating HOXA7, thereby restoring cisplatin sensitivity (198, 199). Additionally, HOTAIR-mediated platinum resistance in OC can also be attributed to the upregulation of HOXA7 and activation of the Wnt/β-catenin pathway (88). These findings point to it as a possible target for re-sensitizing OC cells to platinum therapy. Besides, Wu et al. demonstrated that the lncRNA WDFY3-AS2 modulates the hsa-miR-139-5p/SDC4 axis and may play an essential role in the platinum resistance of OC (200). Taken together, the above studies show that it is promising to prevent or overcome OC resistance by targeting ncRNAs.

### 3.4 Epigenetic Therapy in Combination With Immunotherapy in OC

Antibodies against inhibitory immune receptors, namely, cytotoxic T-lymphocyte-associated protein 4 (CTLA-4/CD152), programmed death protein 1 (PD-1/CD279), and programmed death ligand 1 (PD-L1/B7H1/CD274), have become effective standard immunotherapy for many advanced malignancies (177, 201). However, its effectiveness has yet to be successfully applied to the treatment of OC (202). Although a small fraction of OC patients respond well to immunotherapy, most patients fail to respond or develop secondary resistance to immunotherapy. Therefore, a combinatorial strategy is needed to enhance the efficacy of immunotherapy for OC, which can be achieved by drug repurposing or *de novo* drug development that stimulates the immune response.

Initial findings showed that epigenetic repressive processes were linked to a “cold” immunological environment in OC. Based on them, epigenetic modifiers were used in several of preclinical and clinical trials to explore immune targeting techniques (188). For instance, Stone and colleagues have demonstrated that DNMTi 5-azacytidine (AZA) activates the type I interferon signaling pathway, boosts IFNγ+ T cells and natural killer (NK) cells, and reduces the percentage of macrophages in the TME. They also discovered that clinically relevant dosages of DNMTi and HDACi increased the responsiveness of OC to immune checkpoint therapy by reducing the immunosuppressive milieu *via* the type I IFN signaling pathway (203). The triple combination of DNMTi/HDACi with the immune checkpoint inhibitor α-PD-1 showed significantly better anti-tumor efficacy than DNMTi 5-azacytidine (AZA) alone or AZA along with HDACi, which may be a therapeutic option for treating OC. In a follow-up study, Travers et al. employed a hypomethylating agent (HMA) in conjunction with 2-difluoromethylornithine (DFMO) to rewire TME in OC, leading to a longer-lasting anti-tumor response. They found that AZA and DFMO, either alone or in combination, significantly recruited CD4^+^/CD8^+^ T and NK cells, decreased tumor burden, and improved the survival of OC patients (204). Another trial used decitabine in conjunction with paclitaxel and carboplatin to treat 55 patients with recurrent OC and found that the triple combination treatment group had a 58% overall response rate, which is superior to other groups (205). Therefore, combinational therapy with epigenetic modifiers may be able to avoid the loss of tumor antigens and expand the T cell pool by recognizing the initially targeted antigens and epitopes, showing a new and intriguing promise for vaccine and cell transfer platforms.

## 4 Conclusions and Perspectives

Although notable progress has been made in treating OC recently, most patients with advanced OC still relapse and eventually die from chemotherapy resistance, notably platinum. In fact, tumorigenesis, progression, and treatment resistance of OC are largely mediated by epigenetic regulation. These epigenetic alterations, such as DNA methylation and histone modifications, may occur before or during drug treatment and develop drug resistance by controlling several essential signaling pathways. Recently, with the improvement of our understanding of cancer-specific epigenetic alterations, targeting the epigenome to prevent or overcome OC drug resistance may be a viable therapeutic option.

In this article, we reviewed how epigenetic modifications play a key role in drug resistance in OC cells by affecting pivotal processes, namely, drug transport and metabolism, downstream signaling pathways, cancer stemness, and the immune microenvironment. Meanwhile, we also highlighted the biological functions of several key epigenetic modifiers, such as DNMT, HDAC, and ncRNAs. As the machinery embroidering epigenetic regulation continues to be deciphered, we conclude that the epigenome may emerge as a novel therapeutic target for OC. In fact, some small-molecule inhibitors targeting epigenetic alterations have entered the stage of clinical trials, which are expected to prevent or overcome OC resistance by changing the drug-responsive epigenome of OC. However, for complex reasons, the response rates of OC to monotherapy are typically modest. Although ongoing clinical trials have shown that epigenetic agents can significantly improve the drug sensitivity of OC when combined with targeted agents (e.g., PARP inhibitors) or immune drugs (e.g., anti-PD-1), leveraging the unique properties of epigenetics to obtain the optimal therapeutic advantage still requires considerable effort in the following research.

## Author Contributions

Conceptualization, CH and LL. Investigation, YW. Writing-original draft preparation, YW and ZH. Writing-review and editing, YW, ZH, and BL. Visualization, YW and BL Supervision, CH and LL. All authors listed have made a substantial, direct, and intellectual contribution to the work and approved it for publication.

## Funding

This work was supported by the National Natural Science Foundation of China (82003113, 82102738, 82103168).

## Conflict of Interest

The authors declare that the research was conducted in the absence of any commercial or financial relationships that could be construed as a potential conflict of interest.

## Publisher’s Note

All claims expressed in this article are solely those of the authors and do not necessarily represent those of their affiliated organizations, or those of the publisher, the editors and the reviewers. Any product that may be evaluated in this article, or claim that may be made by its manufacturer, is not guaranteed or endorsed by the publisher.

## Glossary

**Table:**

OC	ovarian cancer
PARP	poly (ADP-ribose) polymerase
lncRNA	long non-coding RNA
miRNA	microRNA
MGMT	O6-methylguanine-DNA methyltransferase
TME	tumor microenvironment
MDR	multidrug resistance
ABC	ATP-binding cassette
EVs	vesicles
CYP	cytochrome P450
VEGF	vascular endothelial growth factor
TCGA	The Cancer Genome Atlas
IGF2BP3	insulin-like growth factor 2 mRNA-binding protein 3
HR	homologous recombination
DNMT1	DNA methyltransferase 1
TMZ	temozolomide
PARPis	Poly-ADP-ribose polymerase inhibitors
FLIP	FLICE inhibitor proteins
DUB3	deubiquitinating enzyme 3
HDACis	histone deacetylase inhibitors
ATG14	autophagy-related gene 14
METTL3	methyltransferase-like 3
OGT	O-GlCNAc transferase
NRF2	nuclear factor erythroid 2-related factor 2
EGFR	epidermal growth factor receptor
NHEJ	non-homologous terminal junction
CSCs	Cancer stem cells
OCSCs	ovarian cancer stem cells
CAFs	Cancer-Associated Fibroblasts
NNMT	nicotinamide N-methyltransferase
APAF1	apoptosis protease activator-1
PFKFB2	Phosphofructo-2-Kinase/Fructo-2,6-Biphosphatase 2
MSCs	Mesenchymal Stem Cells
TAMs	tumor-Associated Macrophages
DNMTs	DNA methyltransferases
MDS	myelodysplastic syndrome
HDAC	histone deacetylase
HGSOC	high-grade serous ovarian cancer
NGS	next-generation sequencing
CTLA-4/CD152	cytotoxic T-lymphocyte-associated protein 4
PD-1/CD279	programmed death protein 1
PD-L1/B7H1/CD274	programmed death ligand 1
AZA	azacytidine
DFMO	2-difluoromethylornithine
